# ^13^C Natural Isotope Abundance in Urothelium as a New Marker in the Follow-Up of Patients with Bladder Cancer

**DOI:** 10.3390/cancers14102423

**Published:** 2022-05-13

**Authors:** Adam Madej, Ewa Forma, Michał Golberg, Rafał Kamiński, Piotr Paneth, Józef Kobos, Waldemar Różański, Marek Lipiński

**Affiliations:** 1 2nd Clinic of Urology, Medical University of Lodz, Pabianicka 62, 93-513 Lodz, Poland; waldemar.rozanski@umed.lodz.pl (W.R.); marek.lipinski@umed.lodz.pl (M.L.); 2 Department of Cytobiochemistry, Faculty of Biology and Environmental Protection, University of Lodz, Pomorska 141/143, 90-236 Lodz, Poland; ewa.forma@biol.uni.lodz.pl; 3 Department of Histology and Embriology, Medical University of Lodz, Zeligowskiego 7/9, 90-752 Lodz, Poland; michal.golberg@stud.umed.lodz.pl (M.G.); jozef.kobos@umed.lodz.pl (J.K.); 4 Institute of Applied Radiation Chemistry, Lodz University of Technology, Zeromskiego 116, 90-924 Lodz, Poland; rafal.kaminski@p.lodz.pl (R.K.); piotr.paneth@p.lodz.pl (P.P.)

**Keywords:** bladder cancer, stable isotope, nitrogen, carbon, sulfur, cell metabolism, IRMS, DFS

## Abstract

**Simple Summary:**

Bladder cancer (BC) is a common urological cancer and has a high incidence of recurrence. Metabolic changes are one of the hallmarks of cancer. Differences in metabolic pathways between normal and cancerous tissues lead to a heterogeneous distribution of natural isotope abundance. We analyzed the isotope ratio of carbon, nitrogen, and sulfur in normal urothelium and bladder cancer samples and correlated these data with clinical parameters. We found that bladder cancers are ^13^C- and ^15^N-depleted when compared to normal urothelium. Furthermore, decreased ^13^C abundance in normal urothelium is correlated with shorter disease-free survival in bladder cancer patients. Determination of the isotopic signature of normal and cancerous bladder biopsies can provide important information about the risk of recurrence and improve patients’ management by avoiding invasiveness and unnecessary diagnostic procedures such as cystoscopy.

**Abstract:**

Bladder cancer (BC) is the most common urological malignancy and has a high incidence of recurrence. BC cells alter their nutrient uptake and metabolic pathways in order to continue the production of sufficient levels of ATP and metabolic intermediates for proliferation and survival. Changes in metabolic pathways regarding the rate of the enzymatic reaction and transport lead to differences in the content of natural isotopes (^13^C, ^15^N, ^34^S) between normal and cancerous tissues. The assessment of the stable isotopes of carbon, nitrogen, and sulfur in normal urothelium and bladder cancer samples was performed using Isotope Ratio Mass Spectrometry (IRMS). The natural abundance of ^15^N and ^13^C was decreased in bladder cancer samples when compared to normal urothelium. No significant correlation was observed in BC specimens depending on the tumor grade and stage. Samples derived from bladder tumors and normal urothelium had a different pattern of ^15^N and ^13^C isotope abundance. Decreased ^13^C natural isotopes in the normal urothelium of BC patients were significantly associated with a shorter DFS. Our results suggest that isotopic analysis of normal urothelium of BC patients can be used to predict bladder cancer recurrence.

## 1. Introduction

Bladder cancer (BC) is the most common malignancy occurring within the urinary tract. Approximately 3% of all new cancer diagnoses and 2% of all cancer deaths are due to bladder cancer [1]. According to GLOBOCAN estimates, about 573,000 new bladder cancer cases and 213,000 bladder cancer deaths occurred worldwide in 2020, with 77% of the total burden occurring in men [2].

Bladder cancer is frequently multifocal and has a high incidence of recurrence [3]. Approximately 75% of BCs are non-muscle-invasive bladder cancers (NMIBCs) [3,4,5]. The remaining 25% of the patients have high-grade, muscle-invasive bladder cancers (MIBC), which spread into deeper layers of the bladder wall or form metastases. Transurethral resection of the bladder tumor (TURBT) is the mainstay therapy for patients with NMIBC, whereas radical cystectomy is the recommended treatment for muscle-invasive bladder cancer [6]. About 10–20% of NMIBCs progress to muscle-invasive bladder cancer. The recurrence rate of NMIBCs treated with TURBT is up to 70–80% depending on the tumor characteristics [3,4,5].

The clinical management of bladder cancer is complex. Hematuria is one of the most prevalent symptoms in the early stage of bladder cancer. However, hematuria has a low specificity as it is a common symptom of urinary tract infections and stone disease. Therefore, white light cystoscopy and urinary cytology are routinely used for BC diagnosis and follow-up, but both methods have poor sensitivity in the detection of preneoplastic lesions and low-grade bladder cancer. Additionally, cystoscopy has some disadvantages such as its invasiveness and high cost. The patients with NMIBC require lifelong regular cystoscopy and urinary cytology to monitor the appearance of cancer recurrence or/and progression, and TUR, which is carried out in recurrent bladder cancer. These factors make the clinical management of bladder cancer one of the most expensive. The European Organization for the Research and Treatment of Cancer (EORTC) criteria are used to divide NMIBC patients into groups with a low, medium, and high risk of recurrence or progression, but these criteria are based on histopathological parameters such as the cancer grade and stage, the number and size of tumors, and the presence of carcinoma in situ (CIS), and do not reflect the behavior of bladder cancers [5,7]. The five-year probability of progression and recurrence increases to 45 and 78%. Several efforts have been made to improve the prognosis. However, a key challenge remains in uncovering biomarkers for the diagnosis of patients with low-grade bladder cancer and to identify patients at a high risk of recurrence and progression [6].

Metabolic reprogramming is one of the well-known hallmarks of cancer. Cancer cells alter their nutrient uptake and metabolic pathways during cancer initiation, growth, and metastasis in order to continue the production of a sufficient concentration of ATP and necessary metabolic intermediates for proliferation and survival [8,9]. Considering the critical role of metabolism in bladder carcinogenesis, an increasing number of studies have been focused on the close implication of metabolism in different aspects of bladder cancer [10,11,12,13]. Numerous metabolic changes have been observed in bladder cancer, including the up-regulation of glycolysis, increased pentose phosphate pathway, glycogen, and lipid metabolism pathways [14]. The cells and tissues of living organisms contain various stable isotopes, such as carbon (^12^C and ^13^C) and nitrogen (^14^N and ^15^N), for which the heavy forms represent about 1.1 and 0.37%, respectively. Changes in metabolic pathways regarding the rate of the enzymatic reaction and transport often led to differences in the content of natural isotopes (^13^C, ^15^N, ^34^S) between normal and cancerous tissue [15]. 

Determination of the metabolic changes, based on the isotope signature of normal bladder epithelium and bladder cancer biopsies, can provide important information about the tumor, the risk of recurrence, and progression, and improve patient management by avoiding unnecessary cystoscopies. Therefore, the aim of our study was to evaluate the isotope ratio of carbon, nitrogen, and sulfur in normal and cancerous bladder tissues using isotope ratio mass spectrometry (IRMS).

## 2. Materials and Methods

### 2.1. Ethical Approval

The investigation was performed with the approval of the Bioethical Commission of the Medical University of Lodz and the National Science Council, Poland (approval No RNN/344/18/KE). The study was conducted in accordance with the guidelines for Good Clinical Practice and in compliance with the 1964 Helsinki Declaration and its later amendments. All patients provided written informed consent before undergoing their procedures. The personal information of the patients was anonymized when necessary.

### 2.2. Patients

The research was carried out on 40 patients with bladder cancer who were treated in the 2nd Clinic of Urology, Medical University of Lodz, Poland with a transurethral resection of bladder tumor (TURBT). The patients were recruited between January and July 2019. Before this, the resection samples of healthy urothelium and tumor tissue (about 2 mm^3^ each) were selected for histopathological and IRMS studies. Samples of normal urothelium were taken from the opposite, normal-looking wall of the bladder. If the tumor was too small for all investigations, samples were not collected. In 27 patients, specimens were taken from the tumor and healthy tissue, in 10 cases only from healthy tissue, and in 3 only from the tumor. After TURBT, the patients were followed-up from 29 to 37 months. During this time, 16 patients had a recurrence of bladder cancer, 5 patients had tumor progression, and 5 patients died. The socio-demographic features of the study subjects are presented in Table 1. Patients from which normal and cancerous bladder tissue specimens originated had the same geographical origin (central Poland, Lodz voivodeship), and thus important differences in the composition of body matter are improbable.

### 2.3. Histological Classification of Bladder Cancer Samples

Histopathological examination was performed on bladder tumor and normal mucosa formalin-fixed paraffin-embedded tissue samples. The material assessment was done based on routine Hematoxylin & Eosin-stained slides. Samples were graded according to the World Health Organization (WHO) Classification of Tumors of the Urinary System and Male Genital Organs in two schemes. Both are based on the level of cells’ differentiation; the first one includes papillary urothelial neoplasm of low malignant potential (PUNLMP), high-grade (HG) or low-grade (LG) papillary urothelial carcinomas (WHO 2004/2016), and the other defines tumors as G1-G3, which stands for well- (G1), moderately (G2) and poorly (G3) -differentiated (WHO 1973). Staging, which is defined by the tumor’s infiltration into lamina propria and muscle layer, was based on the up-to-date TNM Stage Classification of bladder cancer. Due to the used specimen collection technique, which was performed with the above-mentioned TURBT, all examined carcinomas were superficial, low-stage tumors. All the samples were reviewed by two experienced pathologists. The clinicopathological characteristics of the bladder cancers are shown in Table 2.

### 2.4. Isotope Ratio Mass Spectrometry

The assessment of stable isotopes was performed by means of Isotope Ratio Mass Spectrometry (IRMS) with the use of a Sercon HS2022 Continuous Flow Isotope Ratio Mass Spectrometer (Sercon Limited, Crewe, England) connected to a Sercon SL elemental analyser (Sercon Limited, Crewe, England) for simultaneous carbon–nitrogen–sulfur (NCS) assessment. Samples for IRMS measurements were fragmented and dried in a vacuum for 5 h. Then, after weighing, about 2 mg material was placed in tin capsules (cylinder, 4 × 6 mm) with a combustion catalyst for sulfur (vanadium pentoxide) for further processing. Material from one specimen was enough to fill from 1 to 3 capsules. As the local-working standard, thiobarbituric acid was used and results were reported in δ-notation versus primary standards (N_2_ in air, PeeDee Belemnite (PDB), and Canyon Diablo Troilite (CDT) for δ^15^N, δ^13^C and δ^34^S, respectively), where δX = (R_sample_/R_standard_ − 1) × 1000. δX represents ratios of N (^15^N/^14^N), C (^13^C/^12^C), and S (^34^S/^32^S) isotopes. The results were expressed in per mille (‰). The precision of measurements for every element was about ±0.1–0.2 δ-unit. When the sample contains less (more) heavy isotopes than the international standard, the δ values are negative (positive).

### 2.5. Statistical Analysis

Since the carbon, nitrogen, and sulfur isotope ratios in normal and cancerous bladder tissue specimens did not follow a normal distribution (the Kolmogorov–Smirnov test), non-parametrical statistical tests (the Mann–Whitney U-test and Spearman rank correlation test) were applied. A one-way analysis of variance was used for the detection of differences in normal and tumor bladder tissue samples (the Mann–Whitney U-test). The Kruskal–Wallis test with post hoc multiple comparisons was used according to clinical data.

Disease-free survival (DFS) was assessed with the Kaplan–Meier method and compared using the long-rank test. A multivariate DFS analysis was performed for all of the variables that were significant in the univariate analysis using the Cox regression model. The disease-free survival curves were compared between two groups: low (≤median value) and high (>median value) δ^15^N, δ^13^C, and δ^34^S values.

The distribution of quantitative variables is represented by the means and standard error of the mean. All *p* values < 0.05 were regarded as statistically significant.

Statistical analysis was performed using GraphPad Prism 5.0 (GraphPad Software, San Diego, CA, USA).

## 3. Results

### 3.1. Natural Abundance of ^15^N, ^13^C, and ^34^S in Normal Urothelium and Bladder Cancer Samples

**Figure 1:**
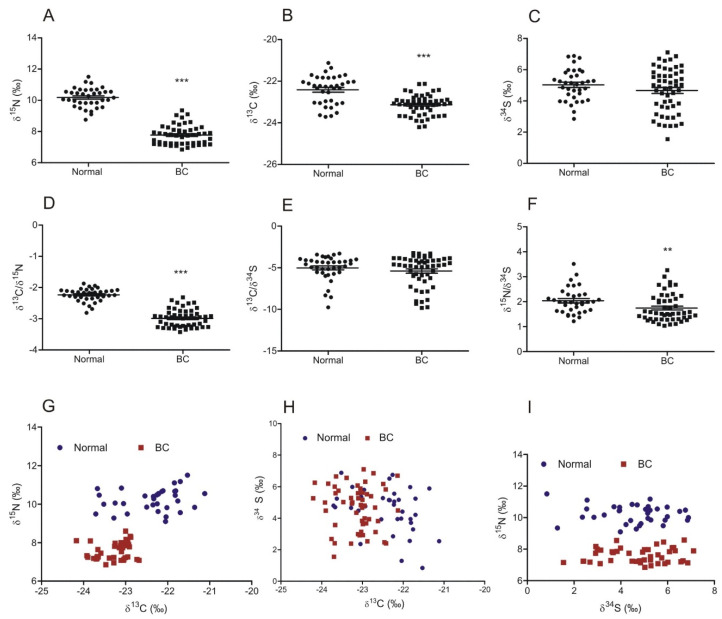
The abundance of natural stable isotopes of nitrogen, carbon, and sulfur in normal urothelium and bladder cancer (BC). Level of δ^15^N (**A**), δ^13^C (**B**)**,** and δ^34^S (**C**) and the ratio of δ^13^C/δ^15^N (**D**), δ^13^C/δ^34^S (**E**)**,** and δ^15^N/δ^34^S (**F**) in urothelium and bladder cancer samples (mean ± SEM). (**G**–**I**) Scatter plots showing the relationship between the abundance of isotope pairs (δ^13^C–δ^15^N, δ^13^C–δ^34^S, and δ^34^S–δ^15^N, respectively) in each sample of normal urothelium and bladder cancer. ** *p* < 0.01; *** *p* < 0.001.

Based on IRMS results, we have observed that δ^15^N for BC was significantly lower than in normal bladder epithelium samples (Figure 1A; *p* < 0.0001), and the assessment of δ^13^C showed that the neoplastic tissues were depleted in ^13^C in comparison to normal bladder urothelium (Figure 1B; *p* < 0.0001). The assessment of δ^34^S has not shown a significant difference in sulfur isotope composition between normal and cancerous bladder tissue samples (Figure 1C; *p* > 0.05). The δ^13^C-to-δ^15^N ratio was significantly lower in BC specimens when compared to normal urothelium (Figure 1D *p* < 0.0001); a similar tendency has been observed for the δ^15^N-to-δ^34^S ratio (Figure 1F; *p* < 0.0001). No correlation has been shown for the δ^13^C-to-δ^34^S ratio (Figure 1E; *p* > 0.05). In order to investigate the relationship between pairs of isotopes in normal and neoplastic bladder tissue specimens, we have prepared scatter plots (Figure 1G–I). Through scatter analysis, we found that bladder cancer and the most normal urothelium samples form distinct populations with significantly different abundances of ^15^N and ^13^C isotopes. However, we observed a population of normal bladder samples with δ^13^C levels that are similar to those observed in cancer (Figure 1G). No significant relationship was found between the abundance of ^34^S and other isotopes (Figure 1H,I). We also did not observe any significant difference between abundances of ^15^N, ^13^C, and ^34^S in normal urothelium and bladder cancer specimens and the socio-demographic parameters of patients, such as age, sex, BMI, occupational exposure, and smoking status.

### 3.2. Level of Natural Isotopes and Histopathological Characterization of Bladder Cancer

**Figure 2:**
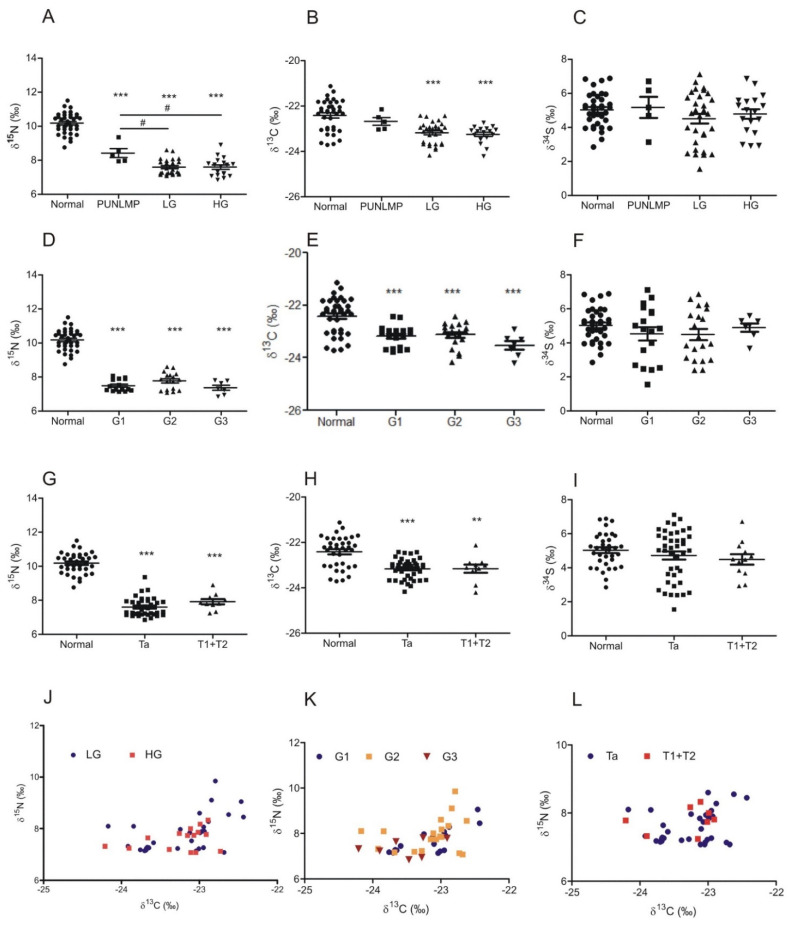
The abundance of ^15^N, ^13^C, and ^34^S isotopes in relation to bladder cancer grade according to the WHO 2004/2016 (**A**–**C**) and WHO 1973 (**D**–**F**) classification systems and TNM stage classification (**G**–**I**) (mean ± SEM). Scatter plots showing the abundance of isotope pairs (δ^13^C—δ^15^N, δ^13^C–δ^34^S, and δ^34^S–δ^15^N, respectively) in each sample of bladder cancer in relation to tumor grade and stage (**J**–**L**). ** (*p* < 0.01) and *** (*p* < 0.001) indicate significant differences when compared to normal tissue; # (*p* < 0.05) show significant differences between PUNLMP and low/high grade (LG/HG) bladder cancers.

For the plots representing the relation between grading and δ^15^N (Figure 2A,D) we have observed that although tumor samples were significantly ^15^N-depleted, the results for LG- and HG-BC were not different. A statistically significant difference in nitrogen isotope composition was observed between PUNLMP vs. LG and PUNLMP and HG (Figure 2A; *p*_Normal_ vs. _PUNLMP_ < 0.001; *p*_Normal_ vs. _LG_ < 0.001; *p*_Normal_ vs. _HG_ < 0.001; *p*_PUNLMP_ vs. _LG_ < 0.05; *p*_PUNLMP_ vs. _HG_ < 0.05). For G1-G3 grading classification, δ^15^N was lower in all grades in comparison to normal bladder mucosa (Figure 2D; *p*_Normal_ vs. _G1_ < 0.001; *p*_Normal_ vs. _G2_ < 0.001; *p*_Normal_ vs. _G3_ < 0.001), but no significant results were observed between each grade (*p* > 0.05). Similar to nitrogen, the relation between grading and δ^13^C showed a depletion in carbon levels in HG and LG tumors, with similar levels observed (Figure 2B; *p*_Normal_ vs. _LG_ < 0.001; *p*_Normal_ vs. _HG_ < 0.001). No significant difference was observed for PUNLMP samples in comparison to normal tissue (*p* > 0.05); comparable results showed the statistical analysis of normal vs. G1-G3 for carbon isotopes (Figure 2E; *p*_Normal_ vs. _G1_ < 0.001; *p*_Normal_ vs. _G2_ < 0.001; *p*_Normal_ vs. _G3_ < 0.001). No significant results of the δ^34^S analysis were seen for either the HG/LG classification (Figure 2C) or G1-G3 (Figure 2F) (*p* > 0.05).

Results were assessed for T parameter in the TNM classification; for δ^15^N we have shown a significant depletion for Ta and T1 + 2 when compared to normal samples, without any demonstrated differences in tumor stages (Figure 2G; *p*_Normal_ vs. _Ta_ < 0.001; *p*_Normal_ vs. _T1+T2_ < 0.001). An identical observation was made for δ^13^C (Figure 2H; p_Normal_ vs. _Ta_ < 0.001; *p*_Normal_ vs. _T1+T2_ < 0.001). No significant correlations with TNM were observed for δ^34^S (Figure 2I). No characteristic distribution patterns were observed for LG vs. HG (Figure 2J), G1-G3 classification (Figure 2K), or TNM (Figure 2J) (*p* > 0.05).

### 3.3. Natural Isotopes’ Abundance in Normal Urothelium as Biomarkers of Recurrence in Bladder Cancer

**Figure 3:**
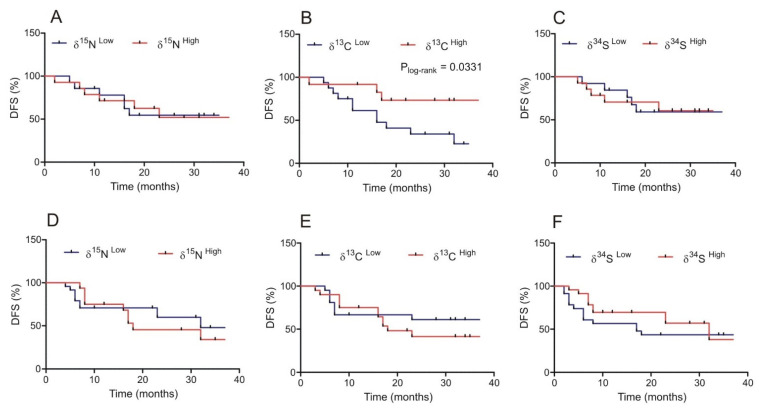
Correlation between the abundance of ^15^N, ^13^C, and ^34^S isotopes in normal urothelium (**A**–**C**) and bladder cancer specimens (**D**–**F**) and disease-free survival (DFS) in patients.

We found that the samples derived from bladder tumors and normal urothelium had a different pattern of ^15^N and ^13^C isotope abundance. Moreover, in the normal bladder epithelium of some patients, we observed a decreased level of the ^13^C isotope, similarly to cancer tissue, with no changes in the ^15^N abundance, which characterized the samples of bladder cancer. Therefore, we investigated the relationship between ^15^N, ^13^C, and ^34^S isotope abundance in normal and cancerous bladder tissue samples and disease-free survival, by Kaplan–Meier survival curve analysis. We observed no statistically significant differences in DFS and the level of natural isotopes in the bladder cancer samples (Figure 3D–F). Interestingly, we found that decreased ^13^C natural abundance in the normal urothelium of bladder cancer patients was significantly associated with a reduced disease-free survival of the BC patients (*p* = 0.0331) (Figure 3B). We did not observe the above-mentioned relationships with regard to ^15^N and ^34^S abundance (*p* > 0.05) (Figure 2A,C).

## 4. Discussion

Bladder cancer cell metabolism is characterized by an increased uptake of glucose, enhanced rates of glycolysis, glutaminolysis, and fatty acids synthesis [8,12,16,17,18,19,20]. The element-forming cells and tissues have different stable isotopes (such as ^13^C/^12^C and ^15^N/^14^N). In metabolic processes taking place within a cell, enzymes preferentially consume substrates containing light or heavy isotopes (isotopologues). This phenomenon is known as the kinetic isotope effect. Therefore, differences in metabolic pathways between normal and neoplastic tissues lead to a heterogeneous distribution of natural isotope abundance [15,21]. 

In our research, we analyzed the abundance of natural nitrogen (^15^N/^14^N), carbon (^13^C/^12^C), and sulfur (^34^S/^32^S) isotopes in normal urothelium and bladder cancer using isotope ratio mass spectrometry. To the best of our knowledge, until now, no studies have been conducted to determine the level of natural isotopes for the assessment of bladder cancer. 

Gender differences in bladder cancer incidence and mortality are related to biologic and epidemiologic factors, as well as socio-economic factors among countries [22,23]. In the case of nitrogen, carbon, and sulfur isotope abundance, we did not observe any gender differences.

We found that bladder cancer was characterized by decreased levels of ^15^N and ^13^C when compared to normal tissue samples. We have also not observed differences in the abundance of these isotopes between low and high grade and stage BC. Analysis of the abundance of natural stable isotopes using IRMS has been performed in other types of cancer, such as oral squamous cell carcinoma, breast cancer, colorectal cancer cells, rhabdomyosarcoma, hepatoblastoma, and nephroblastoma. Bogusiak et al. [24] found that oral squamous cell carcinomas specimens were characterized by a decreased δ^15^N value and an increased δ^13^C value when compared to healthy tissue samples, whereas, breast cancer biopsies were significantly ^13^C-enriched and tended to be ^15^N-depleted. Triple negative (ER-, PR-, HER2-) breast cancer specimens also tended to be relatively ^15^N-depleted. Moreover, ^13^C-abundance in breast cancer specimens was correlated with an alteration in total lipid content [25]. Changes in natural stable isotopes (^15^N and ^13^C) were also observed in solid tumors in children [26,27,28]. The embryonal hepatoblastoma was characterized by a higher abundance of ^15^N than the fetal subtype of this tumor [27]. Similar differences in the nitrogen isotopes’ ratio were observed in the case of the embryonal and alveolar types of rhabdomyosarcoma [26]. Nephroblastoma (Wilms’ tumor) tended to be ^15^N-enriched and ^13^C-depleted in comparison with the normal kidney cortex. Taran and colleagues [28] showed statistically significant differences in δ^15^N and δ^13^C values between histological types of nephroblastoma. High-stage nephroblastoma (3–5 stage) was ^13^C-enriched and ^15^N-depleted. Additionally, the blastemal type of nephroblastoma with focal anaplasia and poorly-differentiated epithelial nephroblastoma were characterized by lower δ^15^N and δ^13^C values than other blastemal and epithelial nephroblastomas, respectively [28]. As the data above, the abundance of carbon and nitrogen isotopes varies depending on the type of tumor. In addition, in the same cancers, the levels of ^15^N and ^13^C change as cancer progresses. 

Sulfur, a major element in the human cells and tissues, is present in two major amino acids, methionine and cysteine, and in aminosulfonic acid, taurine [29]. However, the correlation between changes in the physiopathological processes in the human body is poorly understood. We found no significant differences in ^34^S abundance in bladder cancer when compared to normal urothelium, whereas the serum and erythrocytes of patients with hepatocellular carcinoma (HCC) were significantly ^34^S-depleted when compared to controls [30]. Moreover, Albalat et al. [29] showed that the abundance of ^34^S in HCC patients was correlated to the albumin level. There were no differences in δ^34^S values in the serum of patients with breast and prostate cancer when compared to control subjects [15,29]. These data suggest that the changes in sulfur isotope abundance in patients with hepatocellular carcinoma are the result of the alteration of the liver’s metabolism of S-containing amino acids, transsulfuration, and methylation reactions, which are especially intense in this organ [15,29,30].

Analysis of the scattering of results in terms of the abundance of nitrogen and carbon isotopes showed that the specimens of normal and neoplastic tissue form separate populations. An interesting observation is a fact that some samples of normal bladder epithelium, which did not show any morphological changes, were characterized by a reduced abundance of the ^13^C isotope, similar to bladder neoplastic specimens, but there were no changes in the ^15^N level in them. Numerous studies have shown the presence of genetic and epigenetic changes not only in bladder tumors but also in areas of the urothelium showing no morphological changes. This phenomenon is known as the field effect and is likely responsible for the multifocal growth pattern of bladder cancer and the high recurrence rate [31,32]. Our results suggest that bladder field effect alterations also include metabolic changes that can be detected very early in carcinogenesis by analyzing the abundance of nitrogen and carbon isotopes in the normal urothelium. In addition, we showed that a decreased ^13^C abundance in the normal urothelium was correlated with a shorter disease-free survival. It has been shown that alteration in the expression of proteins involved in carbon metabolism has a negative impact on disease-free survival and overall survival in bladder cancer [10,11,12]. Finally, it should be mentioned that there are limitations to our study. In order for the analysis of the abundance of carbon and/or nitrogen isotopes to be used as a prognostic factor, studies should be carried out on a larger group of patients with bladder cancer. We did not observe any differences in the isotope level depending on the grade and stage of tumor. This may be due to the low sample size of patients with the most advanced bladder cancer. Further studies are needed to confirm the usefulness of isotope abundance analysis as a prognostic factor in clinical practice.

## 5. Conclusions

In our study, we showed for the first time significant differences in the abundance of ^15^N and ^13^C between normal and cancerous bladder tissue specimens. In addition, we found that the depletion of ^13^C in the normal urothelium of bladder cancer patients is correlated with a shorter DFS. Our results suggest that isotopic analysis of normal urothelium of BC patients offers new perspectives on the prediction of bladder cancer recurrence. However, this potential marker needs to be validated as an independent predictor of recurrence in prospective studies.

## Figures and Tables

**Table 1:** Socio-demographic characteristics of study subjects.

Variable	Cases n (%)
**Age** (**years**)	
≥60–<70	11 (27.5)
≥70–<80	18 (45.0)
≥80–<90	9 (22.5)
≥90	2 (5.0)
**Sex**	
Male	33 (82.5)
Female	7 (17.5)
**BMI** (**Body mass index**)	
Normal range (18.5–24.9)	7 (17.5)
Overweight (25.0–29.9)	21 (52.5)
Obese Class I (30.0–34.9)	12 (30.0)
**Occupational exposure**	
Yes	15 (37.5)
No	25 (62.5)
**Smoking status**	
Current	13 (32.5)
Former	24 (60.0)
Never	3 (7.5)

**Table 2:** Histopathological characteristics of bladder cancer.

Variable	Cases n
**Grading** (1973 WHO classification system)	
G1	7
G2	12
G3	5
**Grading** (2004/2016 WHO classification system)	
PUNLMP	4
LG PUC	15
HG PUC	11
**Staging** (2017 TNM classification)	
Ta	23
T1	7
T2	2
**Prior recurrence status**	
Primary	18
Recurrent	12

PUNLMP—papillary urothelial neoplasm of low malignant potential; LG PUC—low-grade papillary urothelial carcinomas; HG-PUC—high-grade papillary urothelial carcinomas.

## Data Availability

Not applicable.
